# Broken Knife Blade Completely Penetrating the Humerus: A Case Report and Literature Review

**DOI:** 10.30476/BEAT.2020.85769

**Published:** 2020-07

**Authors:** Mauricio Gonzalez-Urquijo, Mario Zambrano-La

**Affiliations:** 1 *Tecnologico de Monterrey, Escuela de Medicina y Ciencias de la Salud, Dr. Ignacio Morones Prieto O 3000* *. * * Monterrey, México. 64710*; 2 *D* *epartamento de Cirugía General. Hospital Metropolitano “Dr. Bernando Sepúlveda”. Adolfo López Mateos No. 4600, San Nicolás de los Garza, Nuevo León, México. 66400*

**Keywords:** Knife wound, Stab wound, Bone injury

## Abstract

We present a case of a 23-year-old male patient who presented with a blade knife completely wedged and penetrated on his humerus after a stab wound to his left upper extremity. On palpation, a foreign body was palpated under the skin on the deltoid area. The blade was stuck in the bone, so the surrounding bone tissue was osteotomised until the blade was released. The patient evolved favorably, and at three months follow up, he has a full functional recovery of his arm. Stab wounds are prevalent in emergency departments; however, stab wounds with bone involvement have rarely been reported in the literature. When encountering a blade stuck in bone tissue, removing the blade while avoiding orthopedic, neurological and vascular injuries should be the main goal of the treatment. To the best of our knowledge, this is the third reported case of an intraosseous foreign body in the humerus secondary to a stab wound.

## Introduction

Penetrating trauma is defined as an injury caused by a foreign object piercing the skin, which damages the underlying tissues and results in an open wound. This type of trauma has been on the rise for the past 40 years, with associated extremity involvement in over 50% of these injuries [1]. In the literature, gunshot wound continues to account for the majority of the penetrating extremity wounds, [2] However, in Mexico, the number of penetrating trauma by a knife is much higher than firearm, and only in our hospital in 2019, we treated more than 150 patients with knife wounds. We present a patient who presented with a blade knife completely wedged and penetrated on his humerus, after a stab wound to the deltoid area. To our knowledge, this is the third report case of an intraosseous foreign body in the humerus secondary to a stab wound.

## Case Report

**Fig. 1:**
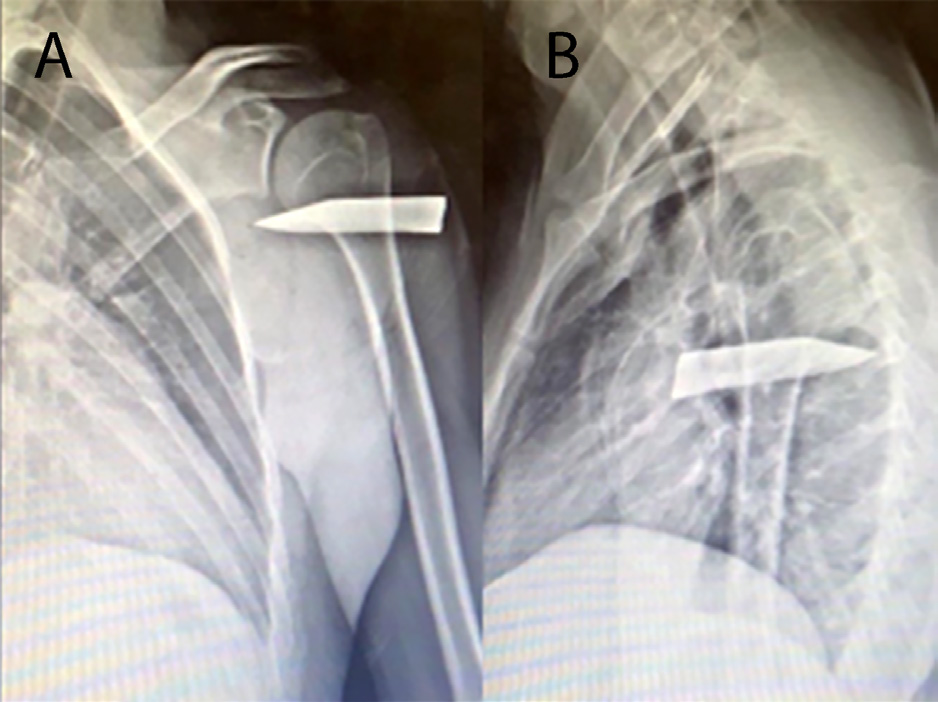
Anteroposterior and later radiography of the humerus demonstrating a foreign body penetrating the proximal humerus

A 23-year-old man presented to the emergency department following an alleged assault with a knife sustaining three stab wounds to his left upper limb. On examination, the patient was hemodynamically stable and afebrile. Physical examination revealed a 6-cm injury over the bicep area and two 2-cm wounds over the deltoid area. On palpation, a foreign body of firm and non-mobile consistency was palpated under the skin on the deltoid area. There was no evidence of a neurological deficit in the left upper limb. Radial and ulnar pulses were with good intensity and good rhythm. Laboratories showed normal hemoglobin of 14.7 g/dl, an elevated white blood count of 14.5×10^^10^, with no other alterations. Chest and left arm radiography were performed on different projections, identifying a broken blade impaled in the humerus bone with the tip of the blade completing penetrating the bone (Figure 1).

**Fig. 2:**
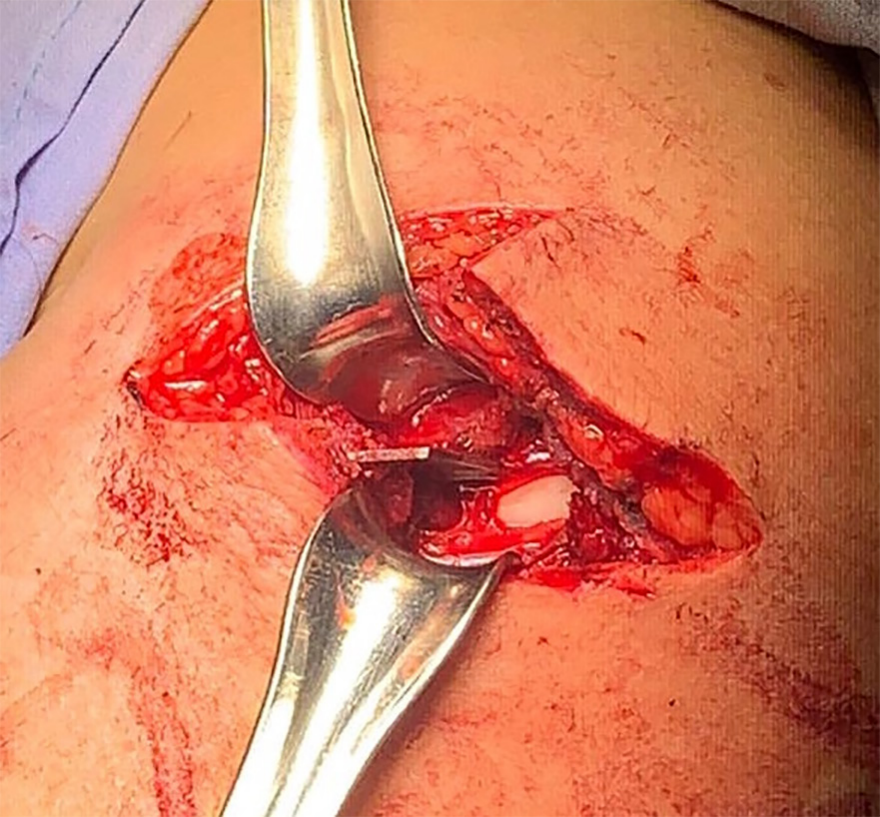
Intraoperative image demonstrating the knife blade penetrating the proximal humerus

**Fig. 3:**
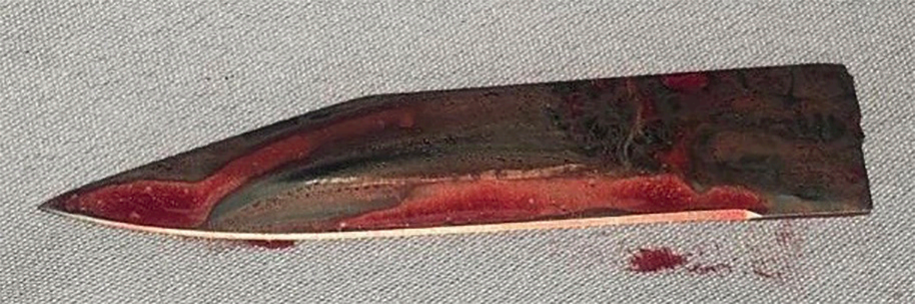
Knife blade after being removed from the patient

**Fig. 4:**
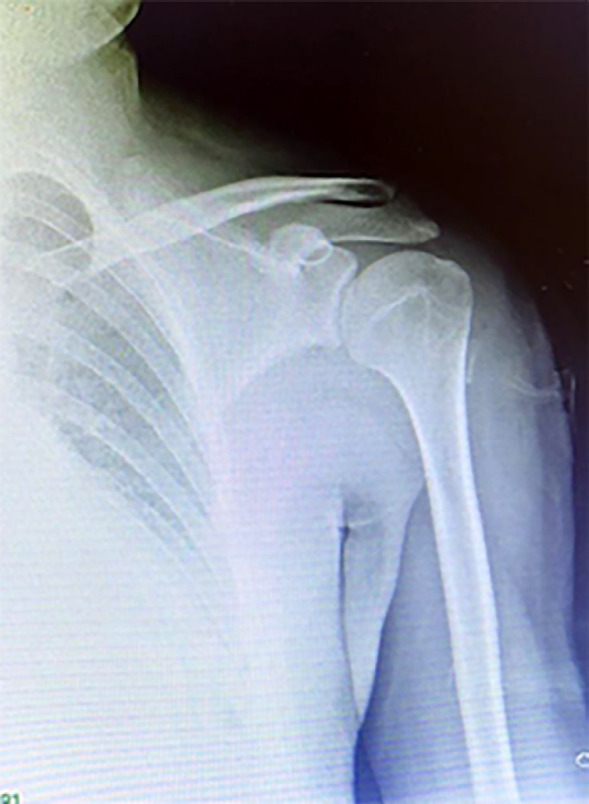
24-hour postoperative anteroposterior humerus radiography showing no bone lesions

The patient was transferred to the operating room. Under general anesthesia, and with the patient in a supine position, we chose to make our approach from the lateral side of the arm, incorporating the original stab wound, allowing circumferential access to the humeral shaft, performing a lateral incision on the deltoid area, extending it proximally and distally, exposing the foreign body entry point. The broken piece of the blade was pressed with the surface of the bone and was strongly fixed in the area of the crest of the greatest tubercle of the humerus **(**Figure 2**). **The humeral artery was unharmed, with an adequate pulse. No lesions to the axillar, radial, or ulnar nerve were seen. Neurological examination of the left upper extremity revealed no motor or sensory deficits. We were not able to pull it out with any surgical instrument, so with the aid of an orthopedic surgeon, the surrounding bone tissue was osteotomised until the blade was released. The blade was then removed (Figure 3). The wound was closed in layers, with a drain inserted below the deltoid muscle for three days, removing it on his fourth postoperative day before discharge. On his first postoperative day an x-ray was taken showing no bone lesions **(**Figure 4). The patient received IV antibiotics and was discharged on postoperative day four with no neurovascular deficit, receiving an oral antibiotic for ten days (cefalotin 500 mg q6h). The arm was placed in a sling for 21 days. At three months follow up, the patient was seen on the ambulatory clinic, with full functional recovery and full pain-free range of movement of his left upper limb. Written informed consent was obtained from the patient for publication of this case report and accompanying images.

## Discussion

Trauma resulting from a knife is due to a dynamic, slow-loaded, compressive force that can damage both hard and soft tissues. Incised wounds are commonly found in defensive positions such as the forearms or upper extremities limbs, as victims attempt to protect themselves [3]. Such as our patient´s injuries, which he referred were caused by a tertiary aggressor. Intraosseous stab wound injuries are rare because of the considerable force needed to impale a sharp object through bone tissue [4]. There is a significant disparity in the forces that could be applied within, and between persons [5]. Hence, it is challenging to evaluate bone wounds sustained by victims systematically. Humphrey *et al*., [3] studied, in experimental models, bone injuries resulting from knife wounds. They concluded that as the length of the bone wound increases, so will the depth and width of that wound. The increasing force increases the extent of the injury as the force acts in the direction of the movement of the blade. 

Following the foreign object removal, our patient was managed following the guidelines, which include foreign body removal, irrigation of the tract, and systemic antibiotics [6]. In penetrating injuries, the breach of the cutaneous barrier and the exposure of underlying tissues to environmental contamination denotes the risk for infection. The consideration of appropriate wound closure involves the grade of tissue devitalization and the age of the wound. If the injury is >8 h old, the risk of infection increases significantly, and closure by secondary or tertiary intention should be considered [1]. In the present case, the patient´s wound had less than 6 hours, so closure of the wound was carried out, giving antibiotics before and after the surgical procedure. 

As there are no more than two reports of retained intraosseous stab wounds in the humerus in the literature, there are no officially documented techniques or clinical guidelines for treating these types of injuries [4,6]. Abboud *et al*., [6] reported a 53-year-old female stabbed on her left arm, with a knife blade lodged in the proximal metaphyseal region of her left humerus, successfully removing it with pliers. Moreover, similar to our case, Quah *et al*., [6] reported a 23-year-old man stabbed in the left arm with a knife. The blade was utterly penetrating and stuck in the humerus. The bone was osteotomised until the blade was released. The two patients evolved favorably, as our patient, with full functional recovery at 6 and 12 months’ follow-up, respectively. 

When encountering this type of injury, a multidisciplinary team must handle the patient, in case a vascular or a neurological lesion is encountered. Proper exposure of the foreign object is necessary, and most likely, the bone will need to be osteotomised, especially in young male’s patients with healthy and strong bones.

In conclusion, this case report shows an interesting and infrequent case of a knife completely penetrated and impaled into the humerus bone, after a stab wound. With the increasing hospital admissions following stab wounds, there is a great probability that physicians will be confronted with similar challenges. Removing the foreign body while avoiding orthopedic, neurological and vascular injuries should be the main goal of the treatment.

## Conflict of Interest:

None.
